# Temperature induces changes in *Drosophila* energy stores

**DOI:** 10.1038/s41598-019-41754-5

**Published:** 2019-03-27

**Authors:** Peter Klepsatel, David Wildridge, Martina Gáliková

**Affiliations:** 1 Institute of Zoology, Slovak Academy of Sciences, Dúbravská cesta 9, 845 06, Bratislava, Slovakia; 2 Stockholm University, Department of Zoology, Svante Arrhenius väg 18B, S-106 91, Stockholm, Sweden

## Abstract

Temperature has a profound impact on animal physiology. In this study, we examined the effect of ambient temperature on the energy stores of the model organism *Drosophila melanogaster*. By exposing adult males to 11 temperatures between 13 °C and 33 °C, we found that temperature significantly affects the amount of energy reserves. Whereas flies increase their fat stores at intermediate temperatures, exposure to temperatures below 15 °C or above 27 °C causes a reduction of fat reserves. Moreover, we found that glycogen stores followed a similar trend, although not so pronounced. To elucidate the underlying mechanism of these changes, we compared the temperature dependence of food consumption and metabolic rate. This analysis revealed that food intake and metabolic rate scale with temperature equally, suggesting that the temperature-induced changes in energy reserves are probably not caused by a mismatch between these two traits. Finally, we assessed the effect of temperature on starvation resistance. We found that starvation survival is a negative exponential function of temperature; however we did not find any clear evidence that implies the relative starvation resistance is compromised at non-optimal temperatures. Our results indicate that whilst optimal temperatures can promote accumulation of energy reserves, exposure to non-optimal temperatures reduces *Drosophila* energy stores.

## Introduction

Temperature affects organisms at all levels of biological organization, from macromolecules to ecosystems^1,2^. These effects are usually explained by dependency of rates of biochemical reactions and biological processes on ambient temperature^3^. Rates of biochemical reactions typically increase exponentially with temperature. This relationship is described by the Van’t Hoff-Arrhenius relation^4^. The temperature dependence of complex physiological and behavioural processes is usually described by a skewed single-peaked curve, termed the thermal performance curve (e.g.^5^).

Metabolism is the sum of all reactions that occur in an organism and provides energy to perform vital processes and to maintain organismal and cellular homeostasis^6^. Metabolic rate is the rate of energy expenditure per unit time^7^. This rate is usually measured indirectly by quantifying the rate of oxygen consumption or carbon dioxide production^8^. Although it is well-known that various physiological processes scale with metabolic rate (e.g.^9,10^), the character of this scaling (isometric/proportional vs allometric/disproportionate) often differs. For example, metabolism and consumption exhibit different scaling with temperature in beetles, spiders^11^ and sea urchins^12^; metabolic rate increases with temperature faster than the rate of consumption. Higher temperatures are therefore associated with relatively less energy available for fitness-related processes such as reproduction or growth^12^.

The energy that organisms obtain from food is partitioned into various components. In its simplest form, an animal’s energy budget depends on a balance between acquired energy and energy spent on biosynthesis (e.g. growth, reproduction, storage), maintenance, or external work (e.g. movement)^13,14^. To survive periods of food scarcity, part of the obtained energy is allocated for energy reserves. In natural habitats with varying food availability, the quantity of energy reserves may be a decisive factor for an animal’s survival^15^. As energy deposition usually occurs only under optimal conditions, the reduction or depletion of an animal’s energy deposits represents an important bioenergetic indicator of physiological stress^16^. Excess energy is stored either as glycogen or fat (triacylglycerols). Due to its higher energy content per unit mass, the fat stores represent the major energy reservoir of the body^17^. In *Drosophila*, it is mainly the amount of fat stores that determines starvation resistance (e.g.^18–22^). Whilst numerous studies examined factors that influence the amount of *Drosophila* energy stores, such as diet or humidity (e.g.^23,24^), the effects of temperature on energy reserves and the underlying mechanisms are rarely studied and remain poorly understood.

A previous study on the effect of three ambient temperatures (18 °C, 25 °C and 29 °C) and short-term heat and cold stress on energy reserves of *Drosophila* males has shown that elevated temperature and thermal stress lead to a significant reduction of lipid stores^25^. In the present study, we further systematically investigated the effect of temperature on energy stores by examining temperature-induced changes in *Drosophila* fat and glycogen reserves over the range of 20 °C (13 °C–33 °C), covering thus the natural thermal range of this species^26^. Based on the concept of energy-limited tolerance to stress^16^, we presumed that intermediate temperatures might allow accumulation of energy stores. On the other hand, at non-optimal conditions (stressful low and high temperatures), additional energy costs or decreased energy acquisition might lead to reduction of energy stores^16^. To test this hypothesis, we first examined temperature-induced changes in energy stores in *Drosophila* males at eleven temperatures (four-day old males were exposed to different temperatures for eight days).

Next, we hypothesised that the temperature-induced changes in energy stores might result from a mismatch between energy expenditure and acquisition, as has been previously reported^11,12^. Thus, we examined the relationship between ambient temperature, metabolic rate (energy expenditure) and food intake (energy acquisition).

Finally, we assessed the effect of temperature on starvation resistance. As the experimental flies were kept under identical conditions (25 °C) prior to starvation, we expected that starvation survival time is determined only by the rate of energy expenditure at a given temperature. Several studies suggested that non-optimal temperatures might significantly influence organismal energy balance, for example, by increasing energy requirements due to elevated maintenance costs (e.g.^16,27^), or by negatively affecting the metabolic efficiency of energy utilization^28,29^, thus we assumed that these factors would have a negative impact on the starvation survival time. In other words, we hypothesised that if survival is energetically more challenging at non-optimal temperatures, then under conditions without any food (i.e. starvation), the same amount of energy reserves might enable survival at sub- or supraoptimal temperatures for a disproportionately shorter period.

Our study shows that the effect of temperature on energy stores, fat stores in particular, in adult *Drosophila* males is best described by a quadratic function with the maximum at intermediate temperatures (approx. 21 °C). Contrary to the previous studies^11,12^, we did not find a clear evidence of a mismatch between energy acquisition via feeding and energy expenditure (metabolic rate) that could explain the observed changes in energy stores. Finally, our analyses of the relationship between temperature and starvation resistance demonstrates that starvation survival time is inversely proportional to temperature. However, the relative starvation resistance does not appear to be compromised by non-optimal temperatures.

## Results

### Temperature induces changes in energy stores

**Figure 1:**
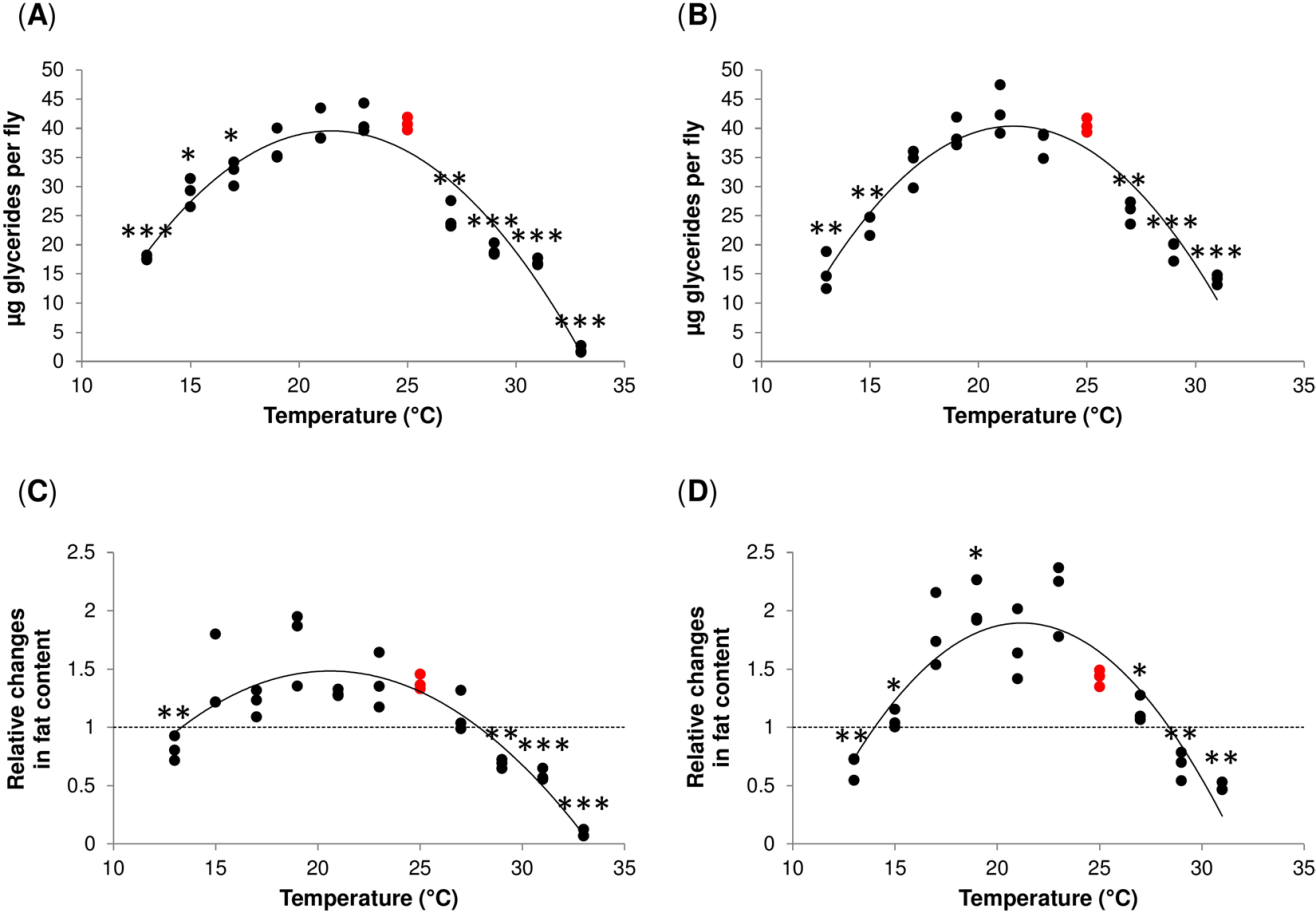
Temperature induces changes in *Drosophila* fat stores. (**A**,**B**) Absolute fat content (µg glycerides per fly) in Oregon R (**A**), and Canton S flies (**B**). (**C**,**D**) Relative changes in the absolute fat content in Oregon R (**C**), and Canton S flies (**D**). The values were calculated as a ratio between the final value and the initial value of fat content for a given biological replicate. Data were compared to the values at 25 °C (red) by Welch’s t-test with the Benjamini-Hochberg correction for multiple testing. Individual data points represent biological replicates. *P < 0.05, **P < 0.01, ***P < 0.001. For global statistical analyses see Supplementary Table S2.

To systematically examine the effect of temperature on the total amount of fat and glycogen stored, we exposed male flies to 11 temperatures between 13 °C and 33 °C. These temperatures are within the natural thermal range of this species^26^. We found that the relationship between temperature and the fat content measured after the exposure to different temperatures for eight days is best described by second degree (quadratic) polynomial function (Fig. 1A,B, Supplementary Table S1). As expected, temperature had highly significant effect on the amount of fat stored (*P* < 0.001) (Supplementary Table S2). In addition, we also detected significant differences between the examined genotypes in the linear (*P* < 0.01) and the quadratic coefficient (*P* < 0.01) of the fitted quadratic functions (Supplementary Table S2). The calculated (optimal) temperature at which the fat content reached maximum is 21.5 °C for Oregon R flies, and 21.6 °C for Canton S flies.

To determine whether the given temperature caused a loss or gain of fat, we also analysed the relative changes in fat content, calculated as a ratio between the final values (i.e. fat content measured after exposure to a given temperature) and the initial values (i.e. fat content measured before exposure to a given temperature) (Fig. 1C,D). This analysis revealed that fat content was significantly reduced at higher temperatures (above 27 °C) and at 13 °C, whereas exposure to temperatures between 17 °C and 25 °C allowed significant fat accumulation in both genotypes.

**Figure 2:**
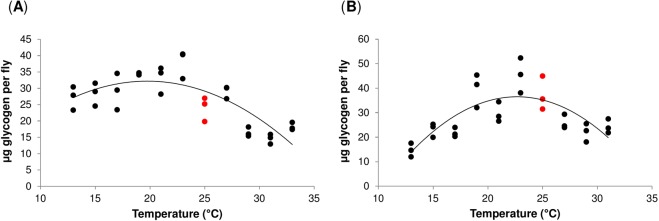
Temperature induces changes in *Drosophila* glycogen stores. (**A**,**B**) Glycogen content (µg glycogen per fly) in Oregon R (**A**), and Canton S flies (**B**). Individual data points represent biological replicates. Data were compared to the values at 25 °C (red) by Welch’s t-test with the Benjamini-Hochberg correction for multiple testing. No significant differences were detected. For global statistical analyses see Supplementary Table S2.

To exclude the possibility that the observed differences in the fat content might be compensated by the glycogen stores, we also examined the effect of temperature on the glycogen content. Interestingly, the glycogen content followed a similar trend with temperature as the fat content, albeit less pronounced; when compared to the values at 25 °C no significant differences in the glycogen content were detected (Fig. 2, Supplementary Table S1). In addition to the significant effect of temperature (*P* < 0.001), there were also significant differences between the genotypes in the linear (*P* < 0.01) and the quadratic coefficient (*P* < 0.01) of the fitted quadratic functions (Supplementary Table S2). The calculated optimal temperature for the glycogen content is 19.8 °C for Oregon R flies, and 22.7 °C for Canton S flies.

### Relationship between metabolic rate, food intake and temperature

**Figure 3:**
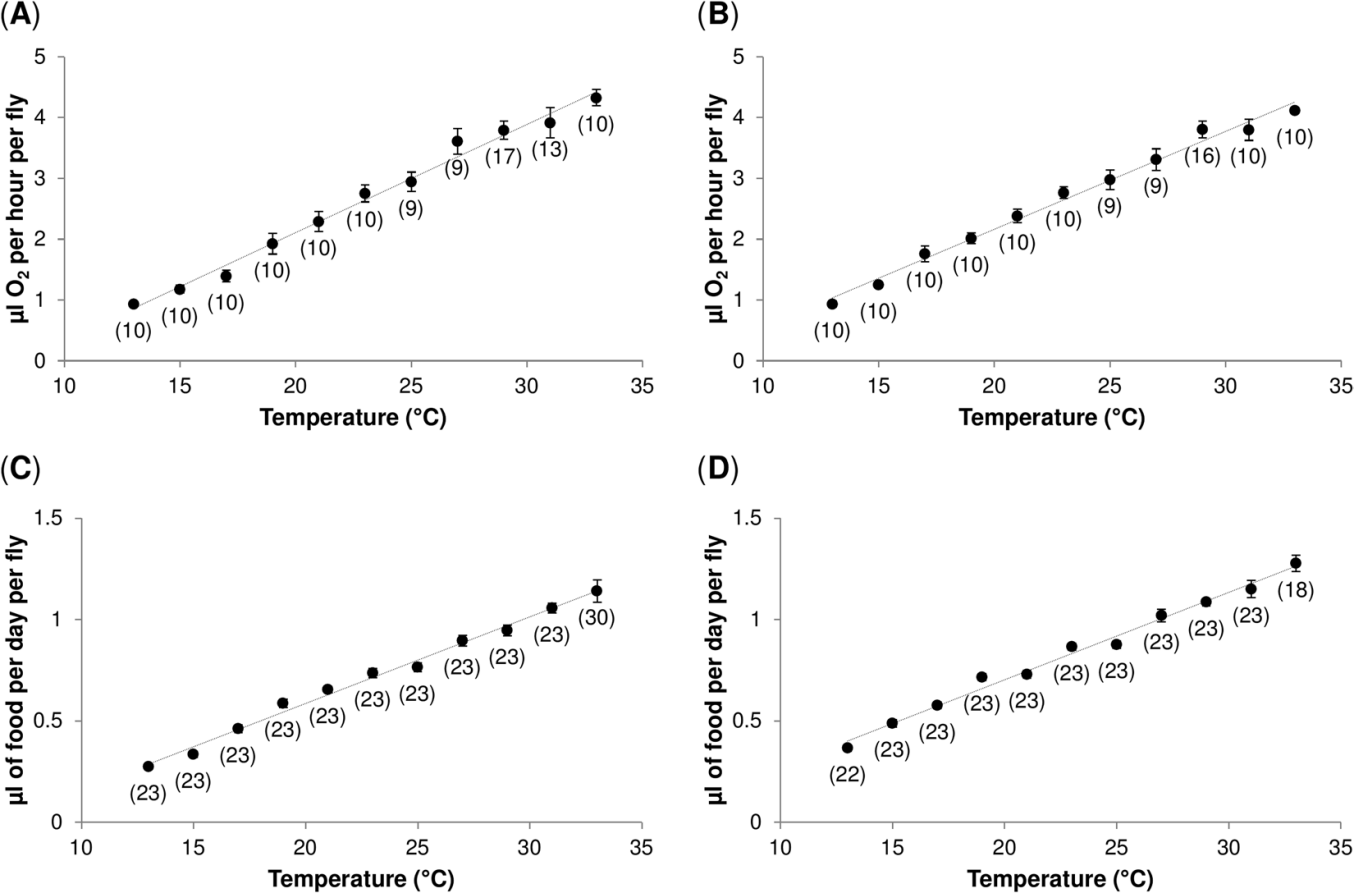
Temperature–dependence of metabolic (**A,B**) and consumption rates (**C,D**). (**A,B**) The relationship between temperature and metabolic rate in Oregon R (**A**), and Canton S flies (**B**). The metabolic rate was measured in males that developed and were kept at 25 °C (12:12 L:D; 60–70% humidity) for four days and were prior to metabolic rate measurements exposed to given temperature for two days to allow acclimation. The values represent mean O_2_ consumption per fly per hour measured during two-hour period (10:00–12:00 AM). (**C**,**D**) The relationship between temperature and consumption rate in Oregon R (**C**), and Canton S flies (**D**). The values represent mean daily food intake of individual males measured over a four day period. For global statistical analyses see Supplementary Table S3. Lines represent linear regressions. Each data point represents the mean value ± s.e.m. Sample size is reported in parenthesis.

Differences in energy stores might arise as a consequence of imbalance between energy intake and energy expenditure. To test this possibility, we measured the metabolic rate (estimated as O_2_ consumption) and individual daily food intake over 20 °C thermal range. As expected, we found a significant positive relationship between temperature and metabolic rate (*P* < 0.001) (Fig. 3A,B; Supplementary Table S3). Although a temperature dependence of metabolic rate is usually described by an exponential function, in the examined thermal range, a linear function fitted our data equally well (Supplementary Table S1). The two *Drosophila* strains did not differ in their metabolic rates (*P* = 0.87), nor in the temperature dependence of these rates (*P* = 0.13) (Supplementary Table S3).

Similar to metabolic rate, the relationship between temperature and feeding was best described by a linear function (Fig. 3C,D, Supplementary Table S1). Both temperature (*P* < 0.001) and genotype (*P* < 0.001) had a significant effect on the daily food intake, with Canton S flies having on average, a slightly higher consumption than Oregon R flies (Supplementary Table S3). However, we did not detect any statistically significant differences between the tested genotypes in the temperature effects on the feeding rate (*P* = 0.88) (Supplementary Table S3).

**Figure 4:**
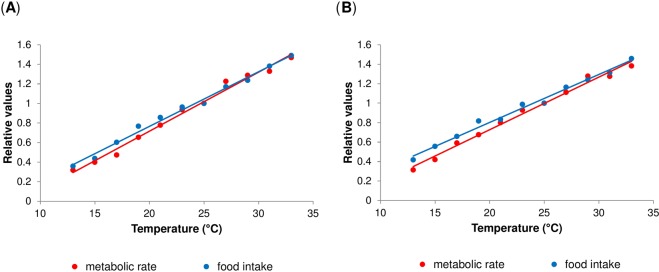
Metabolic and consumption rates scale equally with temperature. (**A**,**B**) Comparison of temperature-dependence of metabolic and consumption rates in Oregon R (**A**), and Canton S flies (**B**). Lines represent linear regressions. Comparison of the slopes either by linear regression (Supplementary Table S4) or by parallelism *F*-test (Oregon R: *P* = 0.12; Canton S: *P* = 0.07) and chi-square test (Oregon R: *P* = 0.95; Canton S: *P* = 0.94) did not reveal any significant differences. Each data point represents the mean value.

Next, we examined whether there are any significant differences in the slopes of the two linear functions that describe the effect of temperature on the relative metabolic rate and the relative food intake (both standardised to the values obtained at 25 °C). Such differences would indicate a potential mismatch between energy intake and energy expenditure. We did not detect any significant difference between the two examined functions for either of the genotypes, i.e. there were no significant differences in the slopes when tested either by linear regression (“trait” by “temperature” interaction: Oregon R: *P* = 0.12; Canton S: *P* = 0.07) or by the parallelism *F*-test (Oregon R: *P* = 0.12; Canton S: *P* = 0.07) and chi-square test (Oregon R: *P* = 0.95; Canton S: *P* = 0.94) (Fig. 4; Supplementary Table S4).

### Temperature dependence of starvation survival time

**Figure 5:**
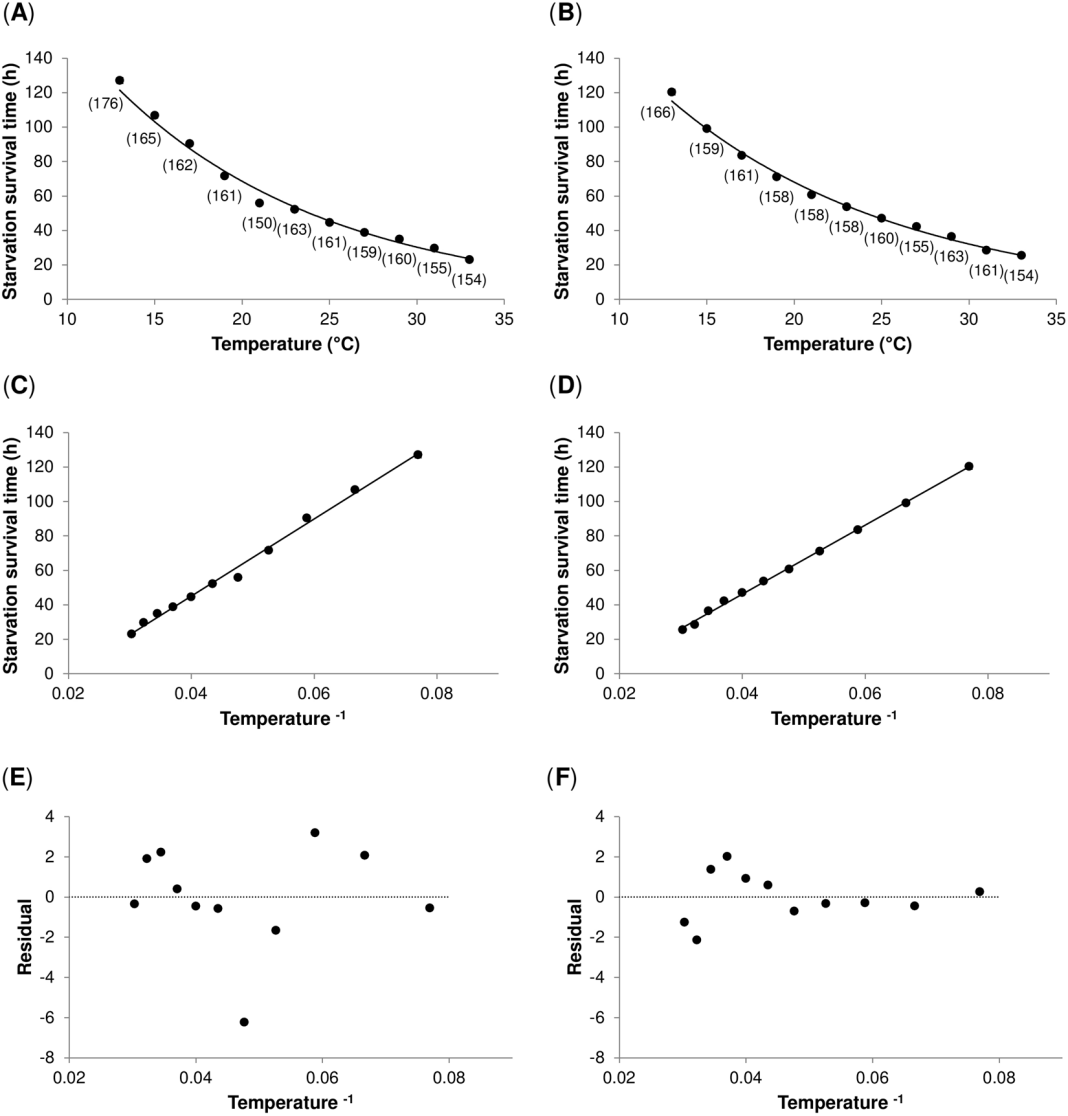
Temperature–dependence of starvation survival time. (**A**,**B**) The relationship between temperature and starvation survival time in Oregon R (**A**), and Canton S flies (**B**) is best described by an exponential function. Starvation survival time was measured in four-day old males that were transferred to 1% agarose (in distilled water) and exposed to eleven different temperatures. Lines represent exponential regressions. Each data point represents the mean value ± s.e.m. Sample size is reported in parenthesis. (**C**,**D**) The relationship between inverse temperature and the starvation survival time in Oregon R (**C**), and Canton S flies (**D**) is best described by a linear function. Lines represent linear regressions. (**E**,**F**) Plot of residuals vs inverse temperature in Oregon R (**E**), and Canton S flies (**F**). The residuals did not depart from 0 in a systemic manner (Shapiro-Wilk W test of normality: Oregon R: *P*-value = 0.08; Canton S: *P*-value = 0.99).

Finally, we examined to what extent starvation resistance is influenced by temperature. First, we analysed the temperature dependence of starvation survival at different temperatures. We found that the starvation survival time is a negative exponential function of ambient temperature (Fig. 5A,B, Supplementary Table S1). Next, to facilitate the visualization and detection of potential deviations in the relationship between temperature and starvation resistance, we linearized the data by plotting the starvation survival time against inverse temperature^30^. We found that this relationship is best described by a linear function (Fig. 5C,D, Supplementary Table S1). This result contradicts the notion of additional energy costs or less efficient use of energy reserves at sub- or supra-optimal temperatures (if the starvation resistance is negatively affected by non-optimal temperatures, a polynomial function would be a substantially better fit for the data). Consistently, the examination of the residuals also did not reveal any obvious non-random pattern (i.e. the residuals did not depart from 0 in a systemic manner), which would otherwise imply that some explanatory information was not captured (Shapiro-Wilk W test of normality: Oregon R: *P*-value = 0.08; Canton S: *P*-value = 0.99) (Fig. 5E,F).

## Discussion

### Thermal reaction norms for energy stores

In this study, we examined temperature-induced changes in *Drosophila* energy stores over a broad range of temperatures (13 °C–33 °C). Consistent with the previous work^25^, we found that elevated temperature substantially depletes fat stores, whereas intermediate temperatures promote fat accumulation. However, we discovered that the changes in fat stores are not a simple linear function of ambient temperature. The effect of environmental temperature on *Drosophila* fat deposits is better characterised by a second-degree polynomial (quadratic) function. This indicates that despite the fact that the flies developed and were acclimated to 25 °C, lower temperatures down to 19 °C enable a comparable or even higher deposition of fat stores than at 25 °C. At temperatures between 15 °C and 19 °C, reduced fat accumulation in comparison to 25 °C could be caused by a substantially lower rate of all biochemical processes. However, further decrease in temperature caused depletion of fat stores. Altogether, these data show that an exposure to non-optimal or stressful low or high temperatures causes significant reduction of lipid stores. Similarly, we also found that the glycogen stores tend to be lower at non-optimal temperatures (although this effect was not statistically significant). Thus the temperature-induced changes in the fat stores are not compensated by carbohydrate reserves. Although we have not measured starvation resistance after exposure to different temperatures, we assume, based on the previous results^25^, that the observed temperature-induced changes in the energy reserves also affect starvation survival. Overall, our findings are in accordance with the concept of the energy-limited tolerance to stress, which posits that depletion of energy reserves can serve as a bioenergetic indicator of adverse conditions^16^.

### Temperature-dependence of metabolic rate and food intake

To elucidate the underlying cause of the temperature-induced changes in energy stores, we examined the rate of energy expenditure (metabolic rate) and energy intake via feeding. As expected, metabolic rate (measured as O_2_ consumption) increased significantly with temperature. Although this relationship is usually described by an exponential (e.g.^3^) or a third-degree polynomial function^31^, we found that within our range of examined temperatures (13 °C-33 °C), a linear function describes the temperature dependence of metabolic rate reasonably well. We assume that the reason for this apparent linearity is that the metabolic rate was measured over relatively narrower (natural) thermal range, which does not include extreme temperatures (see also^28,31^). Other factors that may potentially bias the metabolic rate measurements and which we did not directly account for are locomotor activity, diurnal rhythm or handling stress^32^. However, the calculated Q_10_ values for metabolic rate (2.0 for Oregon R and 2.3 for Canton S flies) in our experiment are consistent with the previous measurements in *Drosophila*^33,34^.

The quantification of daily food intake revealed that temperature has a significant positive effect on consumption rate. The temperature dependence of food intake was best described by a linear function. This result is consistent with previous findings that the food consumption rate in ectotherms increases with temperature (e.g.^35–37^; but see also^11,38^). Lemoine *et al*.^39^ studied the temperature dependence of the food intake in 14 herbivore species from three insect Orders (Lepidoptera, Coleoptera, Hymenoptera). These authors observed that the overall consumption increases with temperature between 20 °C and 30 °C. However, with a further temperature increase above 30 °C, some of the examined species had either declining or increasing consumption rates. Clearly, there exists considerable interspecific variation in the relative food intake at elevated temperatures^39^.

The metabolic theory of ecology posits that the rate of consumption is scaled with metabolic rate^9^. Our results are consistent with this prediction. Within the experimental temperature range, the standardised food intake is linearly scaled with the standardised metabolic rate. In contrast, Rall *et al*.^11^ documented in several predatory beetle and spider species that ingestion rate is less influenced by temperature than metabolic rate; however, the precise relationship between temperature and ingestion rate depends on the prey size. Similarly, Lemoine and Burkepile^12^ found by studying the sea urchin *Lytechinus variegatus* at five temperatures (20 °C, 23 °C, 26 °C, 29 °C, 31 °C) that consumption and metabolic rates are differently scaled with temperature. Specifically, metabolic rate increased more rapidly with temperature than food intake. However, this mismatch was observed only at higher temperatures (above 26 °C). At lower temperatures, consumption was scaled equally with metabolism^12^. We presume that similarly as in the case of consumption rate, there might be a substantial variation in this scaling relationship, which could be caused by interspecific differences in the physiological impacts of stressful temperatures.

### Potential causes of temperature-induced changes in *Drosophila* energy stores

The fact that we did not detect a clear mismatch between the thermal dependence of metabolic rate and food intake might suggest that the observed temperature-induced changes in energy stores could have other cause(s). In general, it is presumed that basal maintenance costs are higher outside of organismal optimal thermal range (e.g.^16,27^). Increased energy flux towards maintenance under non-optimal conditions is explained by additional costs, which include higher protein turnover, production of heat shock proteins, and damage repair (e.g.^1,27^). Such extra costs could be potentially covered by increased utilization of energy stores^25^. Kristensen *et al*.^40^ showed, for example, that *Drosophila* reared at 31 °C have (in comparison to flies reared at 25 °C) downregulated proteins associated with carbohydrate metabolism, energy production and transport, and upregulated proteasome proteins involved in repair or removal of damaged proteins. This might mean that upregulation of the proteasome occurs at the expense of basal metabolic functions^40^. Similarly, Schou *et al*.^41^ found by analysing metabolic profiles that flies that developed at low or high temperatures have a reduced quantity of sugars and metabolites related to energy metabolism. The authors interpreted these changes as “a response characteristic of costs of homeostatic perturbations”^41^. Additional costs might arise also indirectly as a consequence of altered efficiency of energy acquisition. In numerous ectotherms, temperature modulates mitochondrial efficiency via influencing the non-ATP producing respiration (e.g.^42–45^). For example, Martinez *et al*.^45^ reported a reduced mitochondrial efficiency in *Manduca sexta* at supra-optimal temperatures. At elevated temperatures, a higher proportion of substrate is utilised without generating ATP, which reduces cellular energy budget and indirectly increases costs of maintenance^45^. Likewise, Malmendal *et al*.^46^ detected elevated concentration of alanine, a major end-product of energetically less-efficient anaerobic metabolism, in heat-stressed *Drosophila*, which might support the notion of increased anaerobic metabolism at high temperatures. However, such effect has not been observed in flies exposed to cold^47,48^. Although an increased anaerobic metabolism might occur close to the upper thermal tolerance in some terrestrial ectotherms, it is probably not the case at low ambient temperatures^49^.

In our study, we also examined the effect of temperature on the utilization of energy stores. Under starvation, energy is acquired solely from stored reserves; any potential differences in the efficiency of food digestion or absorption (assimilation efficiency) do not play a role. We therefore reasoned that if non-optimal temperature had a negative impact on the metabolic efficiency of energy utilization, this would negatively affect the starvation survival. However, our data on the relationship between temperature and starvation tolerance do not suggest that this would be the case. We found that starvation survival time is inversely proportional to temperature, and there is no evidence that would indicate that non-optimal temperatures disproportionally reduce starvation survival time. Thus, the reduction of energy stores under non-optimal temperatures is most likely not caused by decreased efficiency of energy utilisation. Therefore we speculate that the temperature-induced changes in energy reserves might be rather caused by the altered efficiency of food digestion and absorption, so-called assimilation efficiency. In other words, the energy gain per unit of consumed food might vary with temperature. This could lead to a temperature-driven energy deficit, which might be balanced by utilization of energy reserves. By measuring assimilation efficiency in larvae of the arctic woolly bear moth (*Gynaephora groenlandica*) at three temperatures (5 °C, 15 °C, 30 °C), Kukal and Dawson^50^ revealed that the assimilation efficiency is highest at the medium temperature. In contrast, the assimilation efficiencies of larvae of various insects^51–53^ and terrestrial salamanders^54^ are inversely related to ambient temperature. Finally, the assimilation efficiencies in numerous insects^55–57^, snails^58,59^, sea urchin *Lytechinus variegatus*^12^ and reptiles^60,61^ are temperature invariant. Whether the assimilation efficiency is affected by temperature in *Drosophila* is not known and requires further study.

One confounding factor of the observed temperature-induced changes in energy stores might be differential rate of aging at different temperatures. As aging negatively affects *Drosophila* fat stores and this age-dependent decline in fat content is accelerated by elevated temperature^25^, the observed reduction of energy stores at higher temperatures might be caused by age-related processes, such as an increased rate of apoptosis or general decline in physiological functions. On the other hand, differences in the rate of aging cannot explain the decline in fat stores at sub-optimal temperatures, although it is possible that the same underlying mechanisms operate in both cases.

## Conclusions

Our work provides the first systematic study of the effects of ambient temperature on the energy stores of the model organism *Drosophila melanogaster*. The analysis of energy reserves within the natural thermal range of this species revealed that the reaction norms for the energy stores are best described by a quadratic function with the maximum at intermediate temperatures. To identify the mechanism behind the temperature-induced changes in fat and carbohydrate stores, we tested the thermal dependence of the key factors defining the energy balance, i.e. energy intake (feeding) and energy expenditure (metabolic rate). Interestingly, we did not find a clear evidence of a mismatch between energy acquisition and expenditure that could explain the observed changes in energy stores. Based on our data on the temperature dependence of metabolic and consumption rates, starvation resistance and the previous study on the cell death of fat body cells^25^, we presume that the alteration in lipid and glycogen stores is caused by interplay between several factors. These might be, for example, differences in assimilation efficiencies (lower at non-optimal temperatures), or the rate of apoptosis of fat body cells (higher at stressful temperatures; see Klepsatel *et al*.^25^). However, the precise factors and the extent to which they affect energy homeostasis at different temperatures remain to be elucidated.

## Materials and Methods

### Fly stocks and maintenance

In this study, we used two wild-type strains of *D. melanogaster*: Oregon R and Canton S. To obtain the experimental flies, 1–2 week old parental flies (approx. 100 individuals) were allowed to lay eggs into vials during a four hour period. To ensure a medium egg density (approx. 150 eggs per 68 mL vial), eggs were counted under a stereo microscope and any superfluous eggs were removed. All experiments were conducted on male flies, which developed at 25 °C (12 h:12 h L:D, 60% humidity) on a standard *Drosophila* medium (6 g agar, 50 g yeast, 50 g sucrose, 70 g maize flour, 5.12 mL propionic acid and 1.3 g methylparaben per 1 L of medium). All flies were transferred every day to vials with fresh medium (approx. 20 mL of medium per 68 mL vial). All described experiments have been approved by the Institute of Zoology, Slovak Academy of Sciences, and performed in accordance with the general ethical guidelines, national law, and the Directive 2010/63/EU (“Directive of 2010/63/EU of the European Parliament and of the Council of 22 September 2010 on the protection of animals used for scientific purposes”).

### Thermal treatments

To systematically examine the effect of temperature on the energy stores, we exposed four-day-old males to 11 temperatures (13 °C, 15 °C, 17 °C, 19 °C, 21 °C, 23 °C, 25 °C, 27 °C, 29 °C, 31 °C and 33 °C) (12 h:12 h L:D, 60% humidity). All experiments were performed in a Binder incubator (Binder constant climate chamber KBF 115); temperatures and humidity were double checked using a digital thermo-hygrometer (Qlog HTL24). For each temperature and genotype, we used three biological replicates (approx. 30 flies per replicate/vial; individual replicates originated from different vials and had different parents). Flies were kept at given temperatures for eight days with daily food exchange (standard medium). Samples (five males per sample; two samples (technical replicates) from each biological replicate) for the lipid quantification were collected at the beginning and at the end of the eight day period of exposure to different temperatures. For the glycogen quantification, we used only the flies collected at the end of the exposure. As Canton S flies did not survive eight days at 33 °C, only Oregon R flies were collected at this temperature.

### Lipid and glycogen determination

Homogenates for lipid and glycogen measurements were prepared as described in Gáliková *et al*.^62^. In brief, five males per replicate (five to six replicates per temperature) were disrupted in 600 µL of 0.05% Tween-20 using TissueLyser II (Qiagen) at 30 s^−1^ for 1 min.; heat inactivated at 70 °C for 5 min and centrifuged at 3000 g for 3 min. Triacylglycerides (TAG) were quantified by coupled colorimetric assay according to Gáliková *et al*.^63^, using the Triglycerides (liquid) assay (Randox, TR1697). Fat content is expressed as absolute fat content (µg glycerides per fly). The value for each biological replicate is expressed as the mean value of the technical replicates. Changes in fat content are expressed as a ratio between the fat content measured at the end *and* at the beginning of the eight day period of exposure to a given temperature (i.e. the final value was divided by the initial value for a given biological replicate). Values >1 indicate an increase in fat content, whereas values <1 mean a reduction of fat stores.

Glycogen content was determined as described in Gáliková *et al*.^63^ by using the GO kit (Sigma, GAGO20)^64^. Glycogen was quantified only in the final samples; glycogen content is expressed as µg glycogen per fly. The value for each biological replicate is expressed as the mean value of the two technical replicates.

### Metabolic rate

The metabolic rate was estimated by the manometric respirometry technique as described in detail in Yatsenko *et al*.^65^. Four-days old males that developed and were kept at 25 °C (12:12 L:D; 60–70% humidity) were prior to metabolic rate measurements exposed to given temperature (12:12 L:D; 60–70% humidity) for two days to allow acclimation. Afterwards, they were placed into sealed chambers and their O_2_ consumption was measured during a 2 h period (10:00–12:00). For each temperature and genotype, at least nine replicates of five males were examined. At the end, all flies were weighed (in a group of five) on an analytical balance (Kern ABS 80-4). The analyses of the mean body weights (one-way ANOVA with Tukey’s HSD (honestly significant difference) post-hoc tests with α = 0.05) did not reveal any statistically significant differences among the flies examined at different temperatures (Oregon: *F*_10,107_ = 0.98, *P*-value = 0.46; Canton: *F*_10,103_ = 1.26, *P*-value = 0.27) (Supplementary Fig. S1), thus metabolic rate is expressed as the mean O_2_ consumption per hour per fly.

### Food intake

Food intake was determined using a modified Capillary Feeder (CAFE)^66^ as described in Gáliková *et al*.^62^. Four-day old males were placed in the CAFE system containing 24 chambers; one chamber remained empty and served as a control for the food evaporation. Altogether, we used 23 males (single fly per chamber) per genotype and temperature. The CAFE system was positioned in a moisture chamber (>90% humidity) and placed in the incubator (12:12 L:D). Capillaries (5 µL Hirschmann micropipettes) with liquid food (5% yeast extract, 5% sugar in water) were exchanged every 24 h. After initial 24 h settling in period, the mean daily food intake was measured over the subsequent four days. Data on the flies that died during the assay were excluded from further analyses. Individual daily food intake (corrected for the food evaporation) was calculated as the average daily food intake of individual flies over the four-day period.

### Starvation resistance assay

Four-day old males (30–35 flies per vial; five vials per genotype and temperature) were transferred to 1% agarose (in distilled water) and exposed to eleven different temperatures. The number of dead flies was recorded every 6 h. Starvation survival time is expressed as the mean time (in hours) till a fly succumbed to starvation.

### Statistics

To estimate the relation between temperature and measured traits, we fitted the measured data (mean values) to different functions using JMP v.13.2.1 software. Based on the Akaike information criterion (AICc)^67^, we selected the function with the lowest AICc value. Specifically, the temperature dependence of fat and glycogen content was best described by a quadratic function, the metabolic rate and food intake by a linear function, and starvation resistance by an exponential function (Table S1).

To compare the temperature-induced changes in the fat content, we performed multiple nonlinear regression analyses with one categorical (“genotype”) and three continuous (“initial fat content”, “temperature”, “(temperature)^2^”) factors, including two interactions (“genotype x temperature”, “genotype x (temperature)^2^”). To compare the temperature-induced changes in the glycogen content, we performed multiple nonlinear regression analyses with one categorical (“genotype”) and two continuous (“temperature”, “(temperature)^2^”) factors, including two interactions (“genotype x temperature”, “genotype x (temperature)^2^”). All pairwise comparisons were performed using the Welch’s *t*-test and *P*-values were corrected for multiple testing using the Benjamini-Hochberg procedure^68^.

Data for the metabolic rate and food intake were analysed using linear regressions with one categorical (“genotype”) and one continuous (“temperature”) factor, including “genotype” by “temperature” interaction. To compare the temperature dependences of food intake and metabolic rate, data were first standardised to the values measured at 25 °C and analysed by linear regressions (using mean values) with one categorical (“trait”) and one continuous (“temperature”) factor, and their interaction (“trait” x “temperature”). For standardisation, we chose the values measured at 25 °C because all experimental flies developed and were kept at this temperature prior to exposure to different temperatures, i.e. the flies examined at 25 °C were the only cohort, which did not experience any temperature shift. To statistically compare the slopes for temperature dependencies, we used the *F*-test and chi-square test for parallelism. Both these tests are based on the extra-sum-of-square analysis, which is a form of ANOVA; to test the null hypothesis the former test uses the *F*-statistic, the latter the chi-square statistic^69^.

All analyses were performed with JMP v.13.2.1 (SAS, Raleigh, NC, USA) and PAST 3.11^70^.

## Supplementary information

Supplementary Information

## Data Availability

The datasets generated during the current study are available from the corresponding authors on reasonable request.
