# Supplementary material for: Temperature induces changes in Drosophila energy stores
Source: Sci Rep. 2019 Mar 27;9:5239. doi: 10.1038/s41598-019-41754-5 (PMC6437209; doi:10.1038/s41598-019-41754-5)
Supplement: Supplementary file 1 — Supplementary Information [file 41598_2019_41754_MOESM1_ESM.pdf]

Supplementary Information

Temperature induces changes in *Drosophila* energy stores

Peter Klepsatel, David Wildridge and Martina Gáliková

Supplementary Figures and Tables

Supplementary Figure S1. Body weight. Comparison of individual body weights of the flies used for the metabolic rate measurements. Data are presented as a mean body weight per individual ± s.e.m. Sample size is reported in parenthesis. Data analyses by one-way ANOVA (with Tukey's HSD (honestly significant difference) post-hoc tests with $\alpha = 0.05$) did not reveal any significant differences among samples (Oregon: $F_{10,107} = 0.98$, P-value = 0.46; Canton: $F_{10,103} = 1.26$, P-value = 0.27).

Supplementary Figure S1.

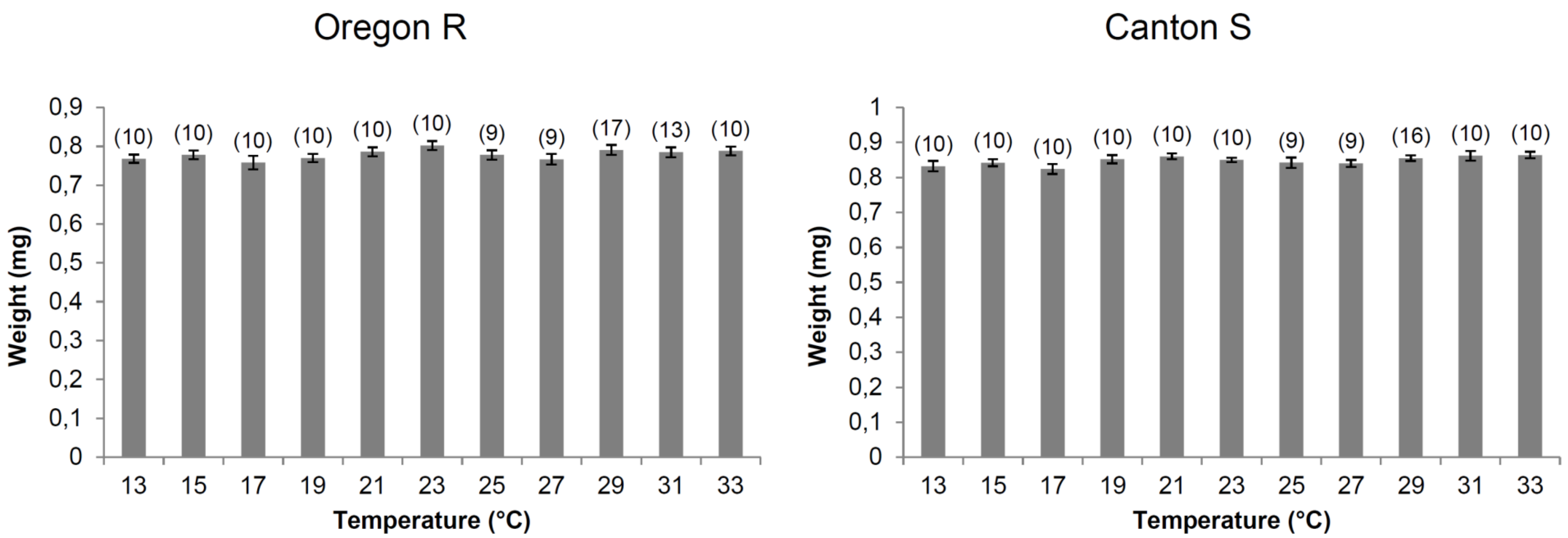

Supplementary Table S1. The Akaike Information Criterion (AICc) and coefficient of determination (R^2) for different functions fitted to data on the temperature dependence of different traits. See Materials and methods for further details.

Trait	Genotype	Linear		Quadratic		Cubic		Exponential	
		AICc	R^2	AICc	R^2	AICc	R^2	AICc	R^2
Fat content	Oregon R	92.64	0.21	**70.37**	**0.94**	77.08	0.94	-	-
	Canton S	84.90	0.02	**64.74**	**0.93**	72.19	0.94	-	-
	combined	172.12	0.14	**127.15**	**0.93**	136.85	0.94	-	-
Relative changes in the fat content	Oregon R	17.82	0.38	**3.49**	**0.90**	10.21	0.90	-	-
	Canton S	26.17	0.08	**12.88**	**0.87**	17.86	0.91	-	-
	combined	40.44	0.25	**9.61**	**0.88**	15.77	0.91	-	-
Glycogen content	Oregon R	78.52	0.40	**76.38**	**0.69**	81.84	0.74	-	-
	Canton S	81.95	0.04	**78.00**	**0.65**	86.89	0.65	-	-
	combined	157.18	0.20	**147.23**	**0.67**	157.55	0.69	-	-
Metabolic rate	Oregon R	**-5.90**	**0.99**	-1.77	0.99	-	-	11.77	0.94
	Canton S	-8.86	0.99	**-11.51**	**0.99**	-	-	10.19	0.94
	combined	**-19.67**	**0.99**	-18.53	0.99	-	-	16.90	0.94
Food intake	Oregon R	**-40.98**	**0.99**	-37.36	0.99	-	-	-22.69	0.96
	Canton S	**-37.94**	**0.99**	-33.46	0.99	-	-	-24.37	0.96
	combined	**-83.82**	**0.99**	-77.81	0.99	-	-	-52.10	0.96
Starvation survival time	Oregon R	89.78	0.92	-	-	-	-	**63.68**	**0.99**
	Canton S	83.87	0.94	-	-	-	-	**55.84**	**0.99**
	combined	169.33	0.93	-	-	-	-	**115.79**	**0.99**
Starvation survival time (vs inverse temperature)	Oregon R	**60.33**	**0.99**	64.74	0.99	-	-	-	-
	Canton S	**43.53**	**0.99**	48.55	0.99	-	-	-	-
	combined	**104.62**	**0.99**	111.44	0.99	-	-	-	-

Supplementary Table S2. Multiple nonlinear (quadratic) regression analyses for the changes in the fat content (µg glycerides per fly) and the glycogen content (µg glycogen per fly). See Materials and methods for further details. *df* - degrees of freedom; SSQ - the sum of squares for each source of variation.

**Trait**	**Source of variation**	***df***	**SSQ**	***F*-ratio**	***P*-value**
Fat content	Genotype	1	1.79	0.15	0.70
	Initial fat content	1	79.03	6.63	0.013
	Temperature	1	5503.73	461.84	<0.0001
	(Temperature)^2	1	6158.22	516.76	<0.0001
	Genotype × Temperature	1	90.08	7.56	0.008
	Genotype × (Temperature)^2	1	93.04	7.81	0.007
	Error	56	667.36	-	-
Glycogen content	Genotype	1	1.67	0.05	0.83
	Temperature	1	1810.38	50.30	<0.0001
	(Temperature)^2	1	1942.77	53.98	<0.0001
	Genotype × Temperature	1	333.10	9.26	0.004
	Genotype × (Temperature)^2	1	267.44	7.43	0.009
	Error	57	2051.44	-	-

Supplementary Table S3. Linear regression analyses for metabolic and consumption rate. *df* - degrees of freedom; SSQ - the sum of squares for each source of variation.

**Trait**	**Source of variation**	***df***	**SSQ**	***F*-ratio**	***P*-value**
Metabolic rate	Genotype	1	0.006	0.03	0.87
	Temperature	1	271.09	1152.19	< 0.0001
	Genotype × Temperature	1	0.54	2.27	0.13
	Error	248	58.35	-	-
Consumption rate	Genotype	1	1.76	105.65	< 0.0001
	Temperature	1	37.21	2238.56	< 0.0001
	Genotype × Temperature	1	0.0004	0.02	0.88
	Error	503	8.36	-	-

Supplementary Table S4. Comparison of temperature dependence of metabolic and consumption rates (based on the mean values) by linear regression analyses for individual genotypes. *df* - degrees of freedom; SSQ - the sum of squares for each source of variation.

**Genotype**	**Source of variation**	***df***	**SSQ**	***F*-ratio**	***P*-value**
Oregon R	Trait	1	0.007	4.28	0.053
	Temperature	1	2.98	1803.41	< 0.0001
	Trait × Temperature	1	0.005	2.75	0.12
	Error	18	0.03	-	-
Canton S	Trait	1	0.02	14.57	0.0013
	Temperature	1	2.35	1685.11	< 0.0001
	Trait × Temperature	1	0.005	3.71	0.07
	Error	18	0.03	-	-
